# Treatment of Pneumonia

**Published:** 1891-03

**Authors:** T. L. Lallerstadt

**Affiliations:** Panola, Ga.


### TREATMENT OF PNEUMONIA.

BY T. L. LALLERSTADT, M. D., PANOLA, GA.

First, hot application to the affected side; opiates in some form, generally the following:

℞ Dovers powders, grs. x.
Cinchonidia sul., grs. xx.

M. ft. caps. No. 6. Sig. One every three to six hours, or morphia hypodermically. Also,

℞ Morphia sul., gr. i.
Fluid ext. gelsemium, gtts. xvi. to xx.
Fluid ext. aconite, gtts vi. to x.
Aqua, ℥ iv.

M. S. Teaspoonful every ten minutes for an hour, and then every hour, while awake, or until patients get easy, then stop. If pains return, or get restless, give as before.

If the bowels have not been acted upon, I would order—provided the patient has never been ptyalized—

℞ Calomel, grs. x.
Aloes, pulv. grs. iii.

M. ft. caps. No. 3. S. One every six hours. If bowels are not moved in six hours after giving last capsule, I would order enema of salts or oil turpentine—prefer former. If these fail, I would introduce a flexible catheter up the rectum and inject a drachm of glycerine, and repeat every thirty minutes until bowels are moved.

When breathing is not good from unaffected lung, I give—

℞ Rhei, pulv. grs. vi.
Valerian, pulv., grs. x.
Oil sassafras, gtts. ii.
Bicarb. soda, grs. x.

M. ft. caps. No. x. S. One every three or six hours.

A case of pneumonia recently under my care, following "La grippe," I ordered first two ℞'s; cough severe and tight, spitting brick-dust sputa, but not suffering much pain. I had a hot iron placed to his side, and in addition to above treatment, commenced giving calcii sulphed granules, one-eighth grain each, one every hour while awake. On my visit the next day, found his cough loose and expectorating freely; no pain or fever; slept very good; skin moist. The following day lung was opening up, bowels open, urine free, eating some and slept well. Treatment continued four days from the commencement of the attack; patient able to sit up, sleeps well, appetite good. I allow my patients to drink and eat whatever suits their fancy

